# The role of stripe orientation in target capture success

**DOI:** 10.1186/s12983-015-0110-4

**Published:** 2015-08-12

**Authors:** Anna E. Hughes, Richard S. Magor-Elliott, Martin Stevens

**Affiliations:** Department of Physiology, Development and Neuroscience, University of Cambridge, Cambridge, UK; Centre for Ecology and Conservation, University of Exeter, Penryn Campus, Cornwall, UK

**Keywords:** Motion dazzle, Motion perception, Camouflage, Visual illusions, Animal coloration

## Abstract

**Introduction:**

‘Motion dazzle’ refers to the hypothesis that high contrast patterns such as stripes and zigzags may have evolved in a wide range of animals as they make it difficult to judge the trajectory of an animal in motion. Despite recent research into this idea, it is still unclear to what extent stripes interfere with motion judgement and if effects are seen, what visual processes might underlie them. We use human participants performing a touch screen task in which they attempt to ‘catch’ moving targets in order to determine whether stripe orientation affects capture success, as previous research has suggested that different stripe orientations may be processed differently by the visual system. We also ask whether increasing the number of targets presented in a trial can affect capture success, as previous research has suggested that motion dazzle effects may be larger in groups.

**Results:**

When single targets were presented sequentially within each trial, we find that perpendicular and oblique striped targets are captured at a similar rate to uniform grey targets, but parallel striped targets are significantly easier to capture. However, when multiple targets are present simultaneously during a trial we find that striped targets are captured in fewer attempts and more quickly than grey targets.

**Conclusions:**

Our results suggest that there may be differences in capture success based on target pattern orientation, perhaps suggesting that different visual mechanisms are involved in processing of parallel stripes compared to perpendicular/oblique stripes. However, these results do not seem to generalise to trials with multiple targets, and contrary to previous predictions, striped targets appear to be easier to capture when multiple targets are present compared to being presented individually. These results suggest that the different orientations of stripes seen on animals in nature (such as in fish and snakes) may serve different purposes, and that it is unclear whether motion dazzle effects may have greater benefits for animals living in groups.

## Introduction

Movement is of key importance in the animal kingdom for many reasons, such as for finding food and mates, and a number of recent studies have considered how motion may have helped to shape animal patterning [1–6]. In particular, it has been suggested that high contrast markings such as those seen on many snakes and fish may act to confuse the speed or direction perception of a predator, reducing the chance of attack or capture and thus increasing an animal’s chance of survival. This is known as the ‘motion dazzle’ hypothesis, in reference to similar patterning that was used on ships in World War I [7, 8].

Research into this area has so far provided mixed support for this hypothesis. Several observational and phylogenetic studies in snakes have suggested that striped banding patterns may create visual illusions when snakes are in motion [6, 9–12] and modelling work has suggested that striped patterns in zebra could interfere with motion detection mechanisms [6]. Some studies have found evidence for stripes or other high contrast patterns providing varied degrees of protection in artificial ‘prey’ capture experiments using human participants, being harder to catch than a range of other pattern types including background matched camouflage targets [1–3]. However, other target types (notably a uniform grey target matching the average background brightness) also appeared to be difficult to catch in these paradigms, casting doubt on a unique benefit of striped patterning. Distortion of speed perception has also been reported for high contrast patterns in human viewers [4]. However, not all research has supported the motion dazzle hypothesis, with some researchers finding less convincing evidence for the benefits of striped targets [5]. In addition, recent phylogenetic studies with zebra have suggested that other factors may have driven the evolution of stripes, including the presence of tsetse flies [13] or thermoregulation [14]. There is therefore still much debate regarding whether motion dazzle could be an explanation for some of the high contrast patterning seen in nature, and if so, exactly what types of pattern cause this effect.

One unresolved issue in the field relates to the effect of stripe orientation. Some research has suggested that targets with stripes parallel to the direction of motion are more easily captured than targets with stripes perpendicular to the direction of motion [5], but other researchers have found no difference in speed perception [4] or capture success [1] between parallel and perpendicular striped targets. From a neurobiological perspective, many neurons that are selective to the direction of motion are also orientation selective, responding only to bars or gratings if they have both their preferred orientation and movement direction (which are usually orthogonal to each other) [15–17]. These results suggest that stripe orientation may have an important effect on the perception of motion.

Psychological research in humans has considered whether there are differences in motion perception between targets (e.g. lines) oriented either in the same direction as the direction of motion versus targets oriented in the opposite direction. However, findings have been mixed and often contradictory; for example, some researchers have found that targets are perceived as moving faster when their orientation is orthogonal to the direction of motion [18, 19], while others have found that they are perceived as faster when their orientation is parallel to the direction of motion [20, 21]. Similarly, discrimination of motion direction has sometimes been found to be best for orthogonal orientations [22, 23] and sometimes best for parallel orientations [24, 25]. Other research has suggested that oblique orientations are perceptually more difficult to process than vertical or horizontal contours [26]. It has been suggested that different types of target orientation might be processed by different mechanisms [27]. Targets oriented parallel to the motion direction have been suggested to be processed by a motion streak mechanism [28], where it is proposed that the slow temporal integration of the motion system may lead to a moving object leaving a ‘smeared’ spatial signal in the direction of motion. This signal can be detected by static orientation detectors and used to help determine the true direction of motion. Meanwhile, targets oriented orthogonal to the motion direction are suggested to be processed using summation of receptive fields of local motion detectors that extract the motion component orthogonal to their orientation [27]. These separate mechanisms might therefore lead to different effects on speed or direction perception for different target orientations.

In the first experiment presented in this paper, we use a capture task paradigm (similar to that used in [3]) where human subjects attempt to catch moving targets sequentially on a touch screen computer to systematically investigate the effect of stripe orientation on capture success. In order to try to resolve the controversies in the motion dazzle literature, we compare targets with stripe patterns both parallel and perpendicular to the direction of motion. In addition, we also test the effect of stripes oriented at oblique angles to the direction of motion, as human psychophysics research suggests that the motion of these targets may be particularly difficult to detect accurately. Our results support the notion that parallel striped targets are easier to capture than perpendicular striped targets, as found in previous studies [5], but do not find support for the hypothesis that oblique stripes are the most difficult to capture compared to other patterns.

In the second experiment, we used the same target types but changed the task that subjects performed; instead of attempting to capture targets one at a time (as has been the case with previous studies e.g. [1–3]), multiple targets appeared on the screen at once and the participant’s task was to attempt to capture all of the targets as quickly as possible. Group movement has long been of interest to biologists. The ‘confusion effect’ suggests that one benefit of group living for animals may be that increasing number or density of prey reduces the ability of a predator to be able to pick out and attack a single member of a group [29]. Several studies in humans and other animals have shown possible benefits of increased group size and/or density in reducing predation [30–33]. It has also been suggested that prey movement patterns may modulate the strength of these confusion effects [30, 32]. However, it has not previously been investigated whether target pattern may affect the strength of confusion. This is particularly relevant as previous modelling work has suggested that since striped patterns are proposed to create misleading motion signals, motion dazzle effects might be stronger with multiple striped targets present [6], and this could make it particularly difficult to pick out one individual in a group of animals all moving at slightly different speeds and directions. In addition, many striped animals (such as zebra and fish) are commonly found living and moving in groups. We therefore hypothesised that the increase in target number might differentially affect the striped targets compared to the non-striped (grey) targets, perhaps making capture more difficult for the striped targets but having no effect on the grey targets. However, the results of this experiment showed both no significant differences between grey and striped targets, but also no differential effects of stripe orientation, in contrast to the findings in Experiment 1.

## Results

### Experiment 1

#### Hits measure

**Fig. 1:**
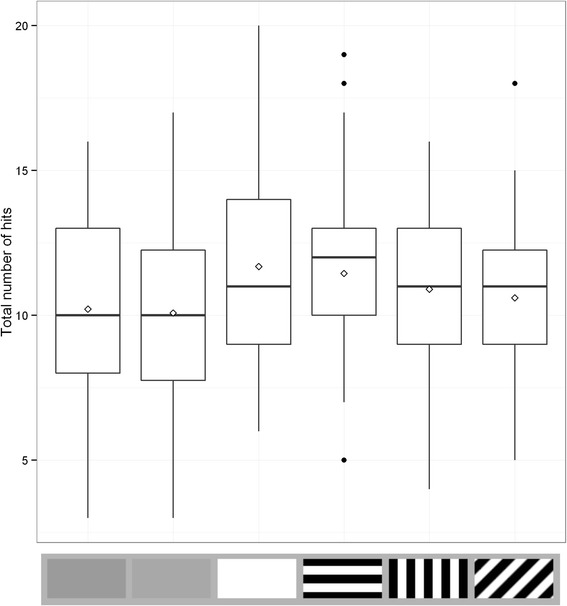
Number of hits for each target type in Experiment 1. Each box contains one value per participant, which is the total number of hits for that target type out of a maximum of 20. Trial types from left to right are average background luminance matching grey, lighter grey, white, parallel stripe, perpendicular stripe and oblique stripe. Whiskers encompass 1.5 x the interquartile range, and points beyond this are plotted as outliers (black circles). Means are represented by white diamonds

All main effects in the simplified hits model (log likelihood = −4708.37, AIC = 9438.74) were significant (target type: χ^2^ = 24.534, d.f. = 5, p <0.001, position group: χ^2^ = 62.025, d.f. = 1, p <0.001, trial number χ^2^ = 39.484, d.f. = 1, p < 0.001). We then compared individual target types to the baseline luminance matched grey target (see Table 1 and Fig. 1 for full results). The lighter grey target was not significantly different in capture rate from the luminance matched target (Z = −0.421, p = 0.674). This suggests that the background luminance matched grey target can be used as a control for the striped targets, even though it has a slightly higher average luminance. The white target was significantly easier to capture than the luminance matched grey target (Z = 3.534, p < 0.001). However, the parallel striped target was also significantly easier to capture than the luminance matched grey target (Z = 2.824, p = 0.005). This is in contrast to the other striped targets, which were both non-significantly different from the grey baseline target (Z = 1.690, p = 0.091 for the perpendicular striped target and Z = 0.794, p = 0.427 for the oblique target). Targets were easier to catch if attempts were made ahead of the midline of the target (Z = 7.876, p < 0.001) and later on in the experiment (Z = 6.284, p < 0.001). Interactions of target type with position group and trial number were non-significant and were dropped during model simplification.

**Table 1:** Full statistical results for the hit rate measure in Experiment 1. These results were obtained using a generalised linear mixed model after model simplification to produce a best fit model. The first five rows detail the planned post-hoc comparisons of the target type, while the final two rows show the effects of the other factors included in the model

Factor	Estimate	Std. error	Z value	p value
Luminance match grey vs. lighter grey	−0.03600	0.08556	−0.421	0.674
Luminance match grey vs. white	0.30490	0.08627	3.534	<0.001
Luminance match grey vs. parallel stripe	0.24293	0.08603	2.824	0.005
Luminance match grey vs. perpendicular stripe	0.14501	0.08580	1.690	0.091
Luminance match grey vs. oblique	0.06801	0.08565	0.794	0.427
Position group	0.54250	0.06888	7.876	<0.001
Trial number	0.16992	0.02704	6.284	<0.001

#### Time to make capture attempt measure

**Fig. 2:**
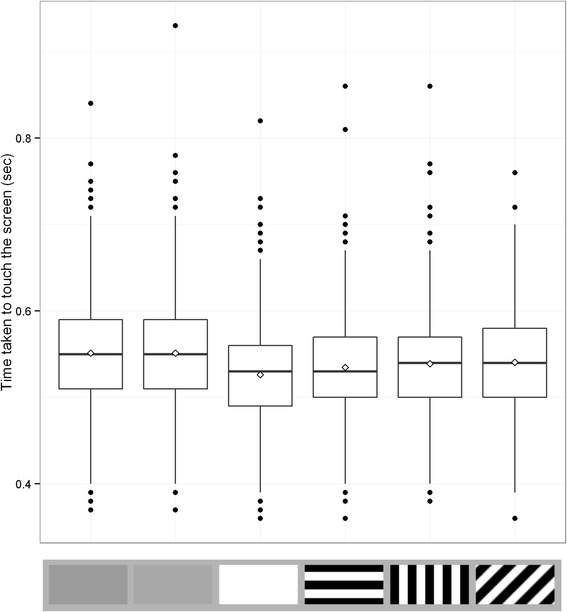
Time taken to touch the screen for each target type in Experiment 1. Each box contains 20 trials per participant. Trial types from left to right are average background luminance matching grey, lighter grey, white, parallel stripe, perpendicular stripe and oblique stripe. Whiskers encompass 1.5 x the interquartile range, and points beyond this are plotted as outliers (black circles). Means are represented by white diamonds

All the main effects in the simplified time model (log likelihood = 7556.904, AIC = − 15087.81) were significant (target type: χ^2^ = 288.09, d.f. = 5, p <0.001, position group: χ^2^ = 131.99, d.f. = 1, p <0.001, trial number χ^2^ = 186.71, d.f. = 1, p < 0.001). When compared to the baseline luminance matched grey target, only the other grey target was non-significantly different (t = −0.002, p = 0.999; see Table 2 and Fig. 2 for full results). All other targets were attempted significantly more quickly (t = −13.772, p < 0.001 for the white target, t = −9.351, p < 0.001 for the parallel stripe target, t = −6.465, p < 0.001 for the perpendicular stripe target and t = −5.212, p < 0.001 for the oblique target). Participants were significantly quicker to make capture attempts ahead of the centre of the target (t = −11.489, p < 0.001) but became slower as the experiment went on (t = 13.664, p < 0.001). As before, interactions of target type with position group and trial number were non-significant and thus were dropped during model simplification.

**Table 2:** Full statistical results for the time measure in Experiment 1. These results were obtained using a linear mixed model after model simplification to produce a best fit model. The first five rows detail the planned comparisons of the target type, while the final two rows show the effects of the other factors included in the model

Factor	Estimate	Std. error	t value	p value
Luminance match grey vs. lighter grey	0.000005920	0.003341	−0.002	0.999
Luminance match grey vs. white	−0.04609	0.003347	−13.772	<0.001
Luminance match grey vs. parallel stripe	−0.03124	0.03340	−9.351	<0.001
Luminance match grey vs. perpendicular stripe	−0.02162	0.03344	−6.465	<0.001
Luminance match grey vs. oblique	−0.01740	0.03338	−5.212	<0.001
Position group	−0.02987	0.002600	−11.489	<0.001
Trial number	0.01526	0.001117	13.664	<0.001

### Experiment 2

#### Total number of attempts to capture measure

**Fig. 3:**
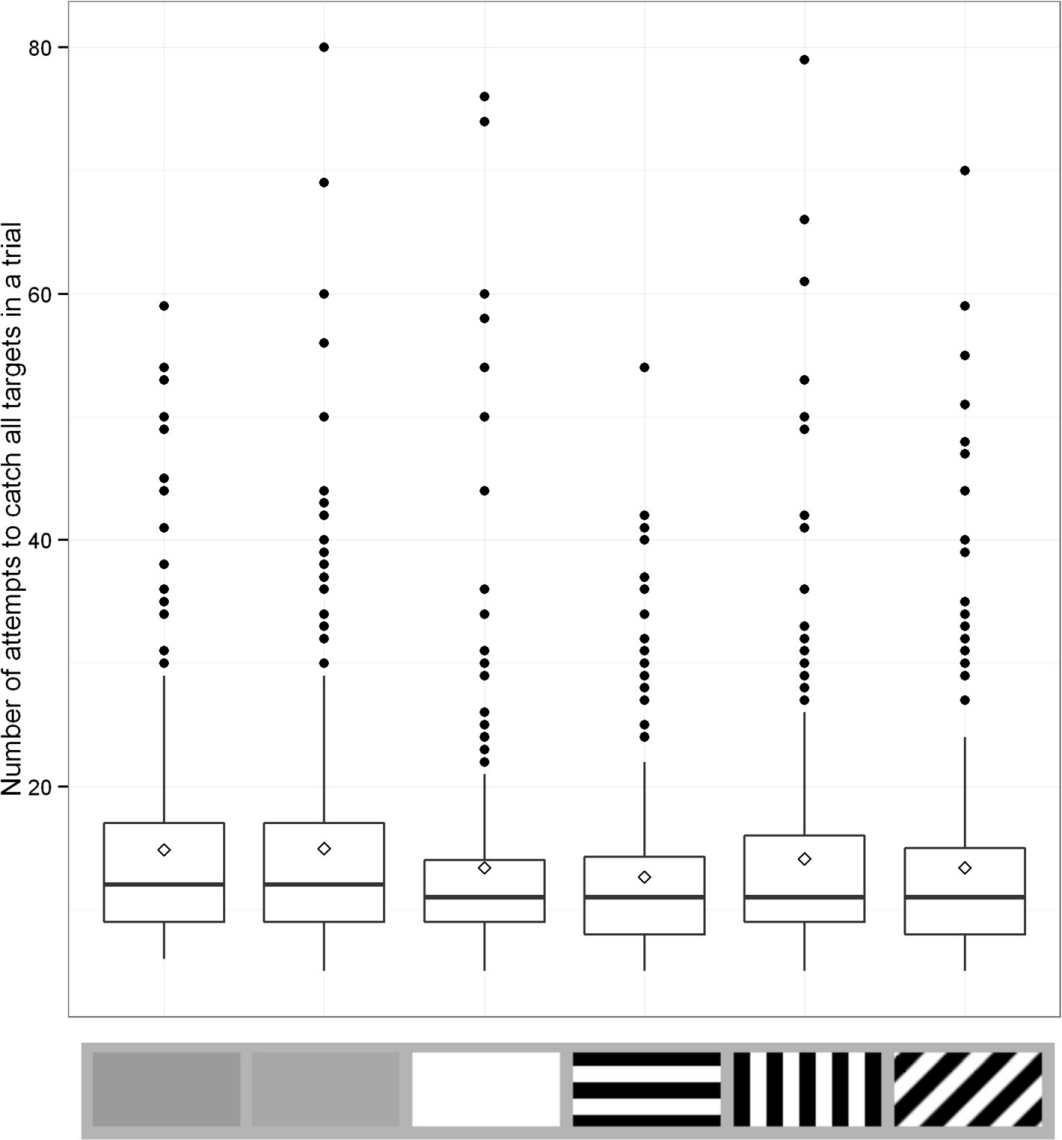
Number of attempts taken to catch all targets for each target type in Experiment 2. Each box contains six trials per participant. Trial types from left to right are average background luminance matching grey, lighter grey, white, parallel stripe, perpendicular stripe and oblique stripe. Whiskers encompass 1.5 x the interquartile range, and points beyond this are plotted as outliers (black circles). Means are represented by white diamonds

Figure 3 shows the distribution of number of attempts to capture for the different target types. In modelling the data, both fixed factors included in the final simplified model (log likelihood = −867.929, AIC = 1757.858) were significant predictors in the model (target type: χ^2^ = 56.118, d.f. = 5, p <0.001 and trial number χ^2^ = 24.144, d.f. = 1, p < 0.001). Table 3 shows the full statistical results, with each target type compared against the baseline luminance matched grey target. This shows that there was no significant difference in the number of capture attempts between the two grey targets (t = 0.002, p = 0.999) but that all other targets had significantly fewer capture attempts (t = −4.136, p < 0.001 for the white target, t = −5.671, p < 0.001 for the parallel stripe target, t = −2.195, p = 0.028 for the perpendicular stripe target and t = −4.226, p < 0.001 for the oblique target). Participants also improved across the experiment, making fewer capture attempts as trial number increased (t = −4.914, p < 0.001), and this improvement was consistent across target types (there was no significant interaction between target type and trial number). These results contrast with those obtained in Experiment 1 for the hits measure, where only the parallel and white targets were significantly easier than the grey baseline to catch.

**Table 3:** Full statistical results for the total number of attempts to capture measure in Experiment 2. These results were obtained using a linear mixed model after model simplification to produce a best fit model. The first five rows detail the planned comparisons of the target type, while the final row show the effects of the other factors included in the model

Factor	Estimate	Std. error	t value	p value
Luminance match grey vs. lighter grey	0.00004098	0.02514	0.002	0.999
Luminance match grey vs. white	−0.1040	0.02514	−4.136	<0.001
Luminance match grey vs. parallel stripe	−0.1426	0.02514	−5.671	<0.001
Luminance match grey vs. perpendicular stripe	−0.05518	0.02514	−2.195	0.028
Luminance match grey vs. oblique	−0.1063	0.02515	−4.226	<0.001
Trial number	−0.06640	0.01351	−4.914	<0.001

#### Total time to capture measure

**Fig. 4:**
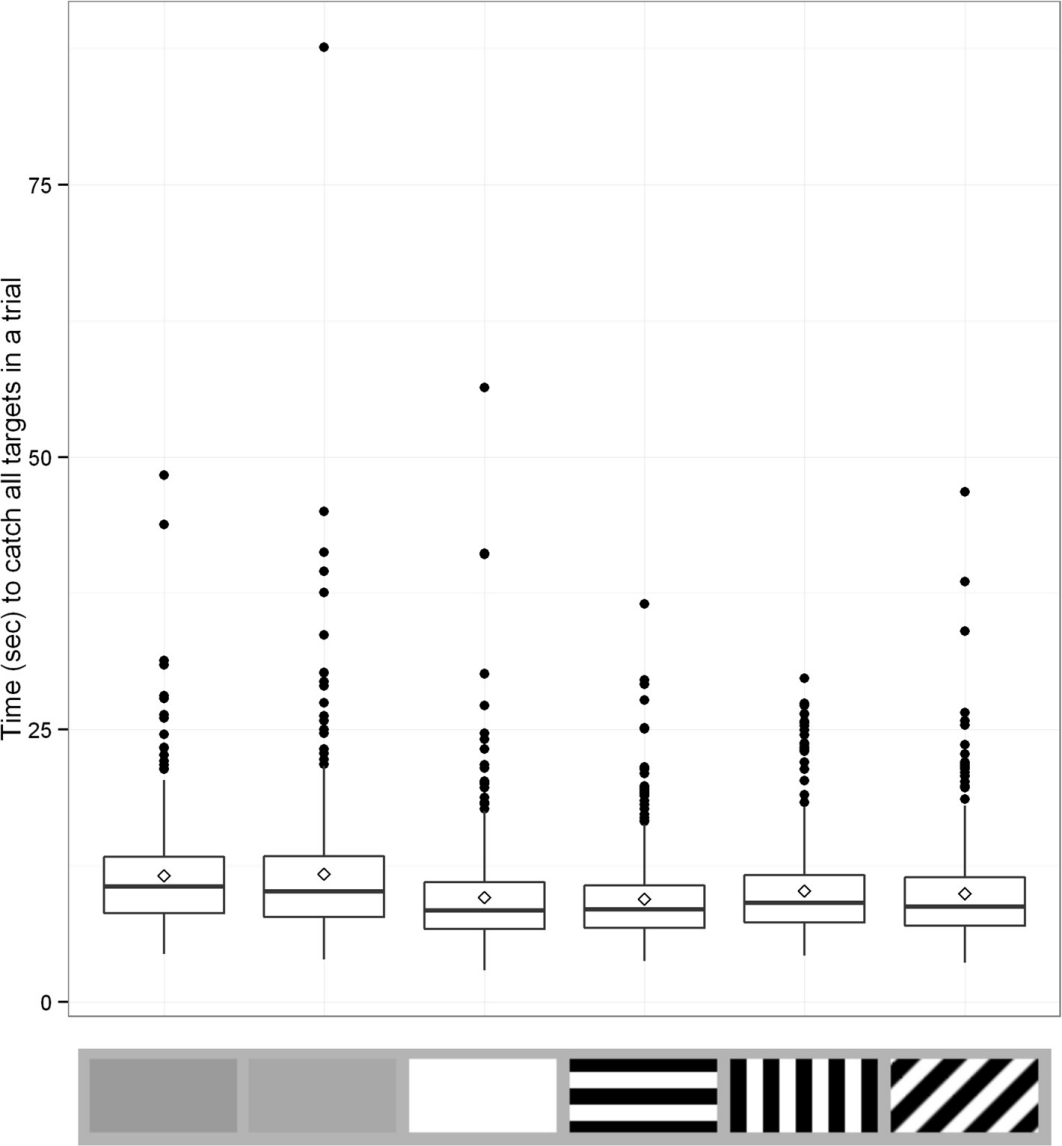
Time taken to catch all targets for each target type in Experiment 2. Each box contains six trials per participant. Trial types from left to right are average background luminance matching grey, lighter grey, white, parallel stripe, perpendicular stripe and oblique stripe. Whiskers encompass 1.5 x the interquartile range, and points beyond this are plotted as outliers (black circles). Means are represented by white diamonds

**Fig. 5:**
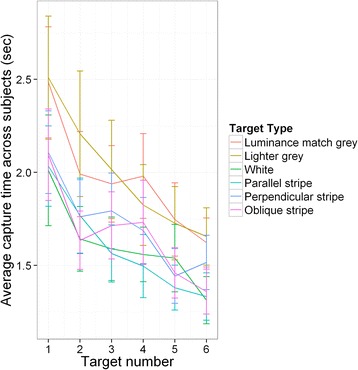
Average time between capture of successive targets in Experiment 2. Results are plotted as a function of target type. Error bars are the standard error of the mean

We also considered the time taken to capture targets (see Fig. 4). All factors remaining in the final model (log likelihood = −12303.47, AIC = 24636.95) were significant (target type: χ^2^ = 203.663, d.f. = 5, p <0.001, trial number χ^2^ = 17.831, d.f. = 1, p < 0.001, and target number χ^2^ = 18.081, d.f. = 1, p < 0.001). Table 4 shows the full model comparisons; it shows that for this measure, the results were very similar to the case where only one target was present, with there being no significant difference in total time to capture all targets between the baseline luminance matched grey and the lighter grey (t = 0.171, p = 0.864), but all other targets being significantly faster to capture (t = −9.870, p < 0.001 for the white target, t = −9.504, p < 0.001 for the parallel stripe target, t = −6.065, p < 0.001 for the perpendicular stripe target and t = −7.500, p < 0.001 for the oblique target). Participants increased in speed across trials as the experiment progressed (t = −4.223, p = 0.001) and also within a trial (t = −4.252, p = 0.013; see Fig. 5). There was no significant interaction between target type and trial number, indicating that there was no differential learning effect across the whole experiment within individual trials. Similarly, there was no significant interaction between target type and target number, suggesting that patterning type did not differentially modulate the difficulty of the task depending upon how many targets were on screen.

**Table 4:** Full statistical results for the total time to capture measure in Experiment 2. These results were obtained using a linear mixed model after model simplification to produce a best fit model. The first five rows detail the planned comparisons of the target type, while the remaining rows show the effects of the other factors included in the model

Factor	Estimate	Std. error	t value	p value
Luminance match grey vs. lighter grey	0.003213	0.01878	0.171	0.864
Luminance match grey vs. white	−0.1854	0.01878	−9.870	<0.001
Luminance match grey vs. parallel stripe	−0.1785	0.01879	−9.504	<0.001
Luminance match grey vs. perpendicular stripe	−0.1139	0.01878	−6.065	<0.001
Luminance match grey vs. oblique	−0.1409	0.01879	−7.500	<0.001
Trial number	−0.06862	0.01625	−4.223	0.001
Stimulus number	−0.04995	0.01175	−4.252	0.013

## Discussion

In this study, we used two different approaches to attempt to determine whether stripe orientation has an impact on capture success. In Experiment 1, we found that parallel striped targets were significantly easier to capture than the grey baseline target, but that this was not the case for the other types of striped target tested in this experiment (perpendicular stripes and oblique stripes). This confirms the results of previous research suggesting that striped and grey targets are similarly difficult to capture in this type of task [1, 3], and also supports work suggesting that parallel striped targets are easier to capture than perpendicular striped targets [5]. We also confirm previous results suggesting that uniform white targets are easier to catch than grey targets [1, 3]. Finally, these results lend some support to the prediction that oblique targets are relatively difficult to capture, although it might have be expected from previous work that they would be harder than both the parallel and perpendicular targets [26]. Experiment 2 asked whether increasing the number of targets presented on the screen affected the difficulty of the different pattern types; we showed that as before, the striped targets were attempted more quickly than the grey baseline, but also that fewer capture attempts were required to catch all the striped targets compared to the baseline grey targets. Experiment 2 therefore failed to replicate the differential capture success seen with the parallel striped target in Experiment 1, and additionally suggests that increasing the number of targets that an observer is viewing does not increase motion dazzle effects.

The finding that the parallel striped target was relatively easy to capture in Experiment 1 supports previous work using a similar paradigm (where subjects were asked to try to ‘hit’ a moving target) that also found parallel stripes were more easily captured than perpendicular stripes [5]. Interestingly, this study also found that parallel striped targets were perceived to be moving more quickly in comparison to a baseline target. As the current results show that in general participants tended to hit behind the target centre on all trials, it could be the case that having an incorrect perception of object speed actually paradoxically improved subjects’ performance on these trials, as they perceived the parallel target to be moving faster than it really was, decreasing the ‘lag’ seen in the responses to other targets. The finding that there are differences in the perception and response to parallel compared to perpendicular targets could also suggest that different mechanisms are implicated in the perception of these targets, with motion streak processes perhaps playing a role for parallel targets and low level motion energy analysis for perpendicular targets [27, 28].

However, it is not clear that this result holds for all cases, as Experiment 2 found no differences between the striped target types; in fact all striped targets were easier to capture than the grey targets. This is in contradiction to the results seen in Experiment 1, where the perpendicular target was not significantly different in terms of capture success. Interestingly, Von Helversen and colleagues also failed to replicate their effects of orientation in a second study, finding instead that the parallel and perpendicular targets were both easier to catch than their baseline target [5], and other research into this area has also been highly contradictory [1, 4, 18–21]. It seems that methodological differences can have a marked effect on the results seen, and this could explain why different results have arisen in the two experiments.

The results of Experiment 2 are particularly surprising as it has been suggested that motion dazzle may be particularly effective in herds [6], and thus we predicted that capture of striped targets would be more rather than less difficult. However, our results suggested that while participants were slower at making capture attempts when there were more targets on screen, this effect was not modulated by target patterning, with all target types showing a similar effect. Unfortunately, we did not collect data that would allow us to test how capture success was affected by target patterning throughout a trial, but it is possible that there was an effect that changed as the number of targets decreased, in a manner that meant no overall effect of pattern type was seen. However, there was no overall differential effect of learning on capture success for different targets throughout the experiment, suggesting that it is unlikely that there are differential learning effects for different target types within trials. We also conducted analysis using a dependent variable of the distance of the successful capture attempts from the target centre (distance of unsuccessful attempts could not be used, as it was not always clear which target participants were aiming for). This analysis found no differential effect of target type, either on its own or in interaction with stimulus number, again suggesting that responses to different target types did not differ markedly as a trial progressed.

One aspect that could explain the results in Experiment 2 is conspicuity, as it is likely that the striped targets are more detectable than the grey targets, perhaps leading to the observed differences in capture rate. However, we argue that it is unlikely that conspicuity underlies the different effects seen in the two experiments presented here. Although this was not explicitly tested in this study, previous work has shown that participants are faster to find striped targets compared to background matching targets when stationary [2]. However, the same study has shown that conspicuity is not necessarily dominant, as striped targets were less accurately targeted when moving [2]. In addition, Experiment 1 of this study shows that there can be differences in capture rate between striped targets that should be equally conspicuous.

It is possible that the results in Experiment 2 could be explained by the fact that this was an extremely crude model of group behaviour, as the targets were not moving together in a group but were instead each following their own random trajectory (although there are certainly cases where animals would flee in a variety of directions when attacked). It could be the case that using more accurate models of joint motion would produce different results to those seen in this experiment. One interesting recent result showed that capture success in a multiple object tracking paradigm using human participants was determined by the interaction between the density of targets and the unpredictability of target motion, with increased density and unpredictability making the target more difficult to catch [32]. This experiment used only one type of (background matched) target and future work could consider whether other patterning types are able to modify this effect.

The result that different orientations of stripes may be captured at different rates, at least under some circumstances, suggests that these pattern types may have evolved for different purposes. Recent phylogenetic analyses in snakes [12] and butterflyfish [34], with both groups containing species with parallel and perpendicular striping patterns, support this conclusion. Allen and colleagues found that parallel striped patterns were associated with rapid escape behaviour, while perpendicular stripes were associated with erratic movements [12]. These findings are particularly interesting given that our results suggest that parallel targets are easier to capture when moving compared to perpendicular stripes. It could be the case that the rapid escape response is an adaptation to try to minimise the effects of parallel striped patterning being easier to capture (as all things being equal, it might be expected that faster animals are harder to catch). This could therefore suggest that parallel stripes have evolved for a purpose unrelated to motion dazzle. However, our results suggest that perpendicular stripes do play a role in making it difficult to accurately track and capture a target. We did not test erratic movement patterns in this experiment, but it would be interesting for future work to consider this, as it might be predicted that the perpendicular striped targets should be even harder to capture relative to the parallel striped targets based on the recent phylogenetic study results [12].

Of course, our experiment is clearly a simplification of the natural situation, and it may be the case that other parameters, such as colour, stripe spatial frequency or the predator’s visual system are critical in determining the efficacy of different pattern types in preventing capture. For example, in butterflyfish and cichlid fish, vertical stripes are associated with particular types of habitat, whereas horizontal stripes are associated with shoaling behaviour [34, 35]. These results suggest that the evolution of these pattern types is complex and associated with many factors, and the interaction with movement may be just one aspect in a larger picture. To fully understand how orientation affects our motion tracking ability, research either needs to focus on exploring a full parameter space of different methodological techniques or needs to consider a specific case based on a real life scenario (for example, designing an experiment where targets and target motion are based on real data for a specific animal, and the subject has the viewpoint of a typical predator). In addition, future work will be required to test potential mechanisms of motion dazzle; the capture measure used in the current study makes it comparable to previous work in this field [1, 2, 5] and provides a measure of the outcome of any dazzle effects, but cannot explicitly test why they occur.

## Conclusions

Our results show that when subjects are attempting to capture individual targets, there may be differences in capture success based on the orientation of stripes on the target, with parallel striped targets being easier to capture than perpendicular or oblique striped targets. However, these differences were not found in a second experiment where multiple targets were present on each trial, perhaps suggesting that the effect of striped patterns is variable depending upon context and that ‘motion dazzle’ may not be strongest in group situations, despite previous predictions. Overall, while motion dazzle remains a plausible explanation for the striped patterns found in a wide range of animal groups, more work is needed to establish its potential value, the factors that make it work, and its ecological relevance.

## Materials and methods

### Experiment 1

**Fig. 6:**
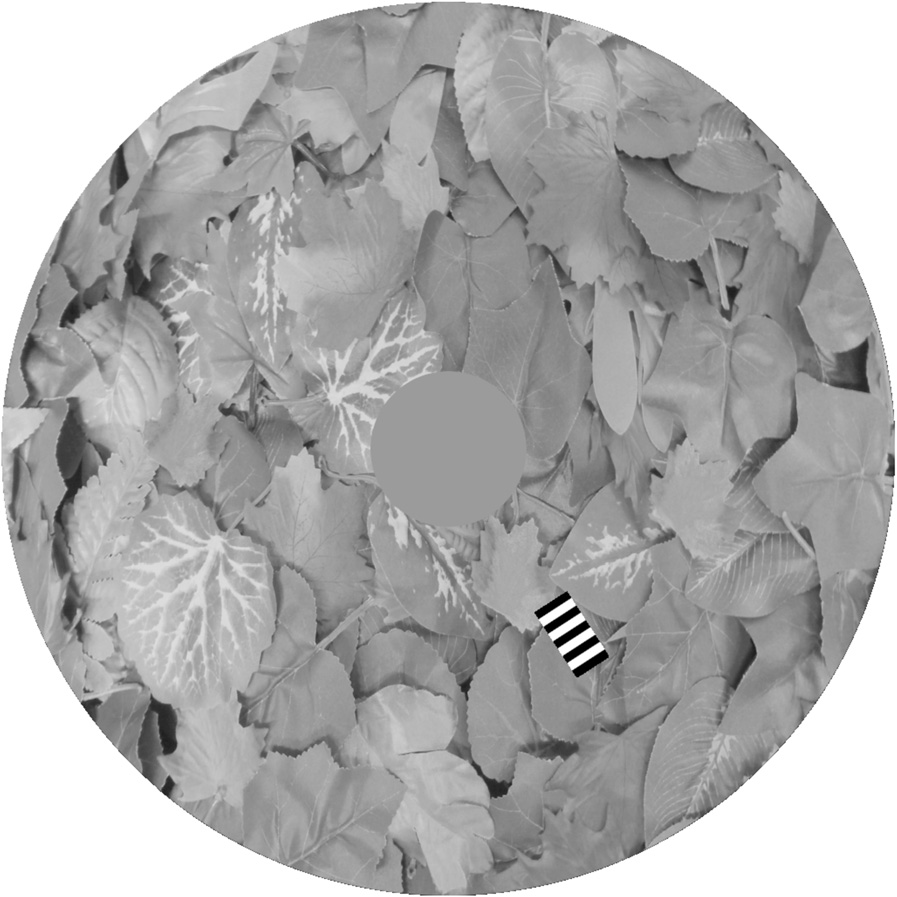
An example screenshot of the general experimental set up of Experiment 1

The experiment was a computer ‘game’ created in Multimedia Fusion 2 (Clickteam 1996–2011) and played on a touch screen monitor (Elo 1515 L; Tyco Electronics, Shanghai, China, 1280 × 1024 pixels, or 42.85 × 34.28 degrees subtended on the viewer’s eye) by human subjects. The achromatic target (90 × 45 pixels, or 3 × 1.5 deg/24 × 12 mm) started behind an occluding circle (diameter 179 pixels, 5.99 degrees) in the centre of the screen. The target then moved out in a random direction at a speed of 20.8 cm/s (approximately 26.7 degrees of visual angle per second) through a circular arena (diameter 1024 pixels, 34.28 degrees) before disappearing. The subjects’ task was to make a capture attempt before the target left the circular arena. The target did not change trajectory once it had started moving. After the subject touched the screen, a cross appeared on the screen with its centre in the position they had clicked. The colour of this cross provided feedback to the participant indicating whether they had hit or missed the target (green and red crosses respectively). The computer program recorded the coordinates of the capture attempt, the coordinates of the target at the time of the capture attempt, the time of the capture attempt and whether the subject had hit or missed the target. After a capture attempt (or after the target had left the screen) there was a short pause before the next target presentation began. The experiment used a block design: each of the six different target types was presented in a random order in one block, and the full experiment contained 20 blocks, meaning that each target type was presented 20 times throughout the experiment at approximately even intervals. Figure 6 shows an example screen shot from this experiment.

**Fig. 7:**
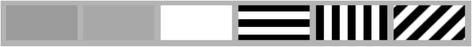
Target types used in both experiments. Trial types from left to right are average background luminance matching grey, lighter grey, white, parallel stripe, perpendicular stripe and oblique stripe

Targets were PNG images, created in Image J. Six different target types were used in this experiment (see Fig. 7). Three targets were created that had different orientations of stripes; either perpendicular to the direction of travel, parallel to the direction of travel or at 45 degrees oblique to the direction of travel. The width of the stripes was matched across targets, with stripes being 10 pixels (0.33 deg) across (and thus the spatial frequency was matched in terms of cycles/degree not cycles/object i.e. the perpendicular target had more cycles of stripes than the parallel target). This was done as it is known that spatial frequency in terms of cycles/degree can affect perceived speed of objects [36, 37], and the design of our stimuli was such that we could not keep both types of spatial frequency constant. Two control grey targets were also created; one with a luminance (perceived brightness) matched to the perceptual midpoint between the white and black stripes (RGB value = 95; see below for details of calibration and how the perceptual midpoint was determined) and one with a luminance matched to the average luminance of the striped targets (RGB value = 113). A white target was also used.

The background exemplars used in this experiment were generated by taking ten photographs of various arrangements of artificial leaves from a fixed height. These images were converted to greyscale and luminance matched to the perceived midpoint between the white and black stripes of the striped targets (RGB value = 95) in MATLAB. This method was used to ensure that lighting conditions and scale were as similar as possible between images. The exact background exemplar presented on each trial was randomised.

### Experiment 2

The target types and background types in Experiment 2 were the same as used in Experiment 1. However, the design of the trial was changed to address the question of whether increasing the number of targets on the screen during each trial would affect capture success differentially for different target types.

**Fig. 8:**
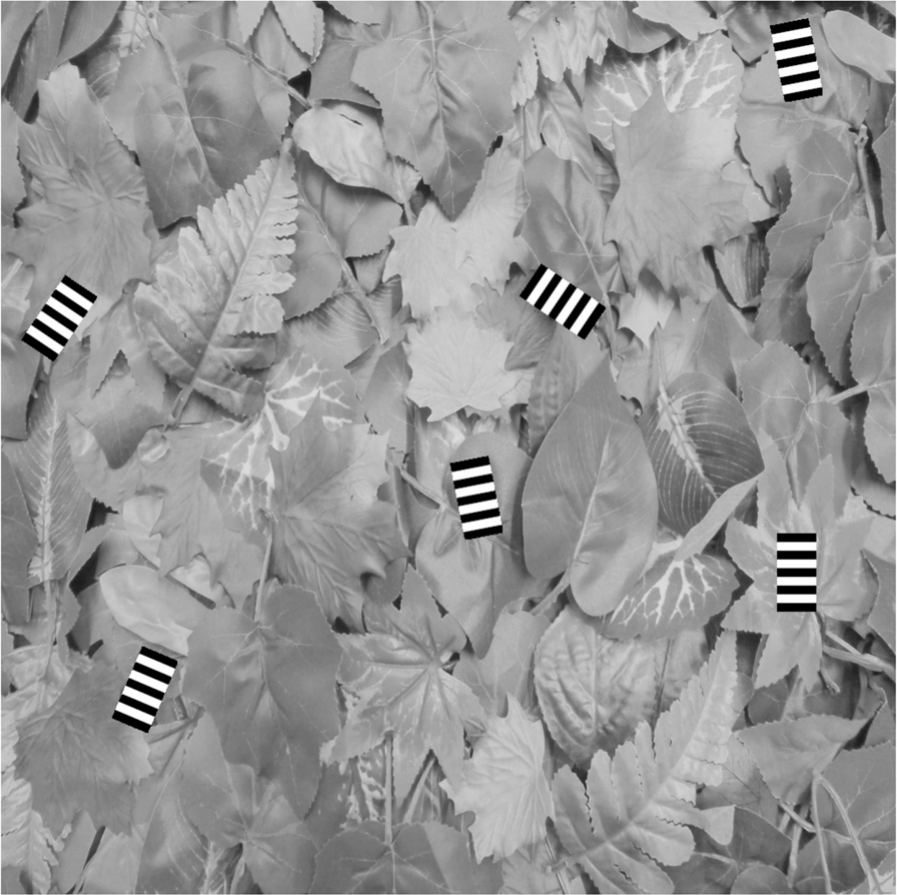
An example screenshot of the general experimental set up for Experiment 2

As in Experiment 1, the ‘game’ was created in Multimedia Fusion 2 (Clickteam 1996–2011). On each trial, a square arena with dimensions 1024 × 1024 pixels (or 34.28 × 34.28 degrees subtended on the viewer’s eye) was presented (see Fig. 2). Six targets of the same type were placed at separate random locations at the beginning of the trial (always at least 100 pixels away from the edge of the arena) and began moving in a randomly selected direction in a straight path (target speeds were equal and identical to those used in Experiment 1). When the target reached the edge of the arena, it would rotate, change direction and continue moving, to ensure that it remained inside the arena and the stripes remained at the same orientation relative to the direction of travel. Targets did not interact with each other (e.g. they slid over rather than ‘bouncing’ off each other). The subject’s task was to attempt to ‘catch’ all six targets as quickly as possible by touching them with their finger. If a target was successfully caught, it disappeared from the screen, and the target and capture positions and time of capture were recorded. The trial continued until all six targets had been caught. The total time taken for the whole trial and the total number of capture attempts (both hits and misses) were then recorded before continuing to the next trial. As in the previous experiments, the trials were presented in blocks, so that in each six trials, all target types were presented. Each participant completed six blocks. Fewer blocks were used in this experiment as each individual trial took longer to complete. 60 subjects took part in this experiment, and none of these had taken part in Experiment 1. An example screen shot of the experimental set up is shown in Fig. 8.

### Monitor calibration

The display was calibrated for human luminance (perceived brightness) perception using a Minolta LS-110 luminance meter (Osaka, Japan) following previous work [1–3]. Images with grey values ranging from 0–255 on an 8 bit scale were displayed on the screen, and the luminance was measured in cd/m^2^ for each image at four different points on the screen and averaged. The grey value was then plotted against the average luminance to determine the value that would represent an intermediate grey between the black and white markings on a ratio scale, and this value was used in target and background creation. The display refreshed at 70Hz, which would equate to a frame by frame displacement of 0.57 degrees. The flicker of the striped targets was 41.6Hz (based on calculating the time taken for one complete cycle of white and black stripes), which was lower than the refresh rate of the display.

### Subjects

60 participants were recruited to carry out each experiment, and each participant only took part in one of the experiments. Subjects were drawn from the undergraduate and graduate populations at the University of Cambridge, were naïve to the experimental aims and were only given enough information to be able to play the game. We did not collect individual age and gender data as they have not been shown to affect results in this type of experimental task, but subjects were predominantly aged between 18 and 25 and both datasets had an approximately even gender split. They gave written consent and the experimental methods were approved by the University of Cambridge Psychology Research Ethics Committee.Viewing distance was approximately constant at 45 cm, and the experiment was conducted in standard laboratory light conditions throughout the working day (lighting levels did not change with time of day as all windows were covered for the duration of the experiment). All subjects received 10 training target presentations first, where a black target was captured on a white background.

### Statistical analysis

Due to the repeated measures design of the experiment, results were analysed using linear mixed models (LMMs) or generalised linear mixed models (GLMMs) [38, 39] using the lme4 package (version 1.1-7) [40] and the lmerTest package (version 2.0-6) [41] in R (version 3.1-0) [42].

For Experiment 1, a model was fitted using target type, trial number and position group (whether the capture attempt was ahead of or behind the midline of the target, as defined by its direction of travel; this factor was included as it greatly improved the model fit, as many more capture attempts were made behind the centre of the target, creating a bimodal distribution) as fixed factors. The initial model also contained all possible first order interactions with target type. The model was simplified based on their AIC weights and log likelihood to produce a best fit model [38, 39]. Analysis was run for Experiment 1 using a hit/miss dependent variable (binomial error structure) and using a time taken to capture measure (log normal error structure). Subject, trial direction, and trial number were included in the hit/miss model as random intercepts; a similar structure was also used for the time measure, but a random intercept of background exemplar was also included (although its variance and standard deviation in the final model was small, and thus we feel that treatment of the background as a random and not a fixed factor is appropriate). Collinearity of response variables was checked using the correlation of fixed effects. We calculated the overall main effects of the models using the Anova function from the car package (version 2.0-20) [43] and then analysed the effects of individual pattern types using planned contrast comparisons [44]. The luminance matched grey target was taken as the reference against which all other targets were compared.

As the design of Experiment 2 differed from that of Experiment 1 (multiple targets were present on the screen at once, instead of a single target on each trial), different measures of capture success were used; analysis focused on the number of attempts taken (log normal error structure) and the length of time taken to capture the targets, as calculated by taking the time since the previous target capture (log normal error structure). Initial models for the total number of hits measure were constructed using the fixed factors of target type and trial number and their interaction, and subject was included as a random intercept and trial number as a random slope. Initial models for the time taken to capture measure included the same fixed factors as the total number of hits model and also a factor indicating which target number (out of the total number of targets on a trial) was being captured and the interaction with target type, to consider whether it was easier to capture the targets when there were fewer of them on the screen, and whether this difficulty was modulated by target patterning. In addition to the random effect structure used for the total number of hits measure, target number was also included as a random factor with trial number as a random slope. Model simplification and inference for both measures was as in Experiment 1.
